# Exploring resilience in family physicians working in primary health care in the Cape Metropole

**DOI:** 10.4102/phcfm.v11i1.1982

**Published:** 2019-10-17

**Authors:** Leigh Wagner, Michael K. Pather

**Affiliations:** 1 Division of Family Medicine and Primary Care, Faculty of Medicine and Health Sciences, Stellenbosch University, Cape Town, South Africa

**Keywords:** work satisfaction, physician well-being, family medicine, resilience, family physicians, primary healthcare practice

## Abstract

**Background:**

Despite the high prevalence of burnout among doctors, studies have shown that some doctors who choose to remain in primary healthcare (PHC) survive, even thrive, despite stressful working conditions. The ability to be resilient may assist family physicians (FPs) to adapt successfully to the relatively new challenges they are faced with. This research seeks to explore resilience through reflection on the lived experiences of FPs who have been working in PHC.

**Aim:**

To explore the resilience of FPs working in PHC in the Cape Metropole.

**Setting:**

The study was conducted among FPs in PHC in the Cape Town metropole, Western Cape province, South Africa.

**Methods:**

A phenomenological qualitative study involved interviewing 13 purposefully selected FPs working in the public sector PHC in the Cape Metropole. Data were analysed using the framework method.

**Results:**

The mean resilience scale was moderate. Six key aspects of resilience were identified: having a sense of purpose, ‘silver lining’ thinking, having several roles with autonomy, skilful leadership, having a support network and self-care.

**Conclusion:**

The aspects that contribute to FP resilience are multi-faceted. It entails having a sense of purpose, ‘silver lining’ thinking, having several roles with autonomy, skilful leadership, having a support network and valuing self-care. Our exploration of resilience in FPs in the Cape Metropole corroborates the findings of previous studies. To ensure physician wellness and improved patient outcomes, we recommend that individual and organisational strategies should be implemented in the absence of long-term policy changes.

## Introduction

Burnout and stress amongst doctors have received considerable attention globally.^1,2,3,4^ Burnout has been defined as a syndrome consisting of emotional exhaustion, depersonalisation and low personal accomplishment.^5^ Burnout negatively impacts both the individual doctor and the healthcare system, resulting in poor-quality patient care, low work satisfaction and even migration of doctors to different work environments.^1,2,6,7^ In the USA, a widely conducted survey found that about one-third to half of the doctors experienced at least one aspect of burnout.^4^

Doctors working in PHC and general practice have been shown higher levels of burnout than those working in other specialities.^3,4^ PHC doctors showed a median duration of practice of 3 years and two-thirds left their PHC practice within 5 years.^8^ South African (SA) studies have shown that junior doctors, particularly community service doctors, have a high level of burnout.^6,9,10,11^ Often, these community service doctors, who are placed in PHC facilities, leave after completion of the compulsory 1-year term – very few return to PHC – thus contributing to the shortage of doctors dealing with PHC.^9,10^ A survey on burnout and depression amongst doctors in PHC in the Cape Metropole found that 27% of doctors had moderate depression and 76% of doctors had high levels of burnout.^6^ It also showed that the longer a primary care doctor was qualified or employed in the primary care setting, the lower the levels of emotional exhaustion and depersonalisation experienced, suggesting that the employment and retention of more senior doctors is required.

Healthcare delivery in SA public sector is based on a PHC approach as adopted by the *Declaration of Alma Ata* in 1978.^12^ However, the legacy of apartheid in South Africa has resulted in inequitable healthcare. All user fees at PHC clinics have been removed since 1997 to improve access to previously disadvantaged communities. Despite the transformation of the healthcare system, access to healthcare still remains a challenge, particularly in rural South Africa.^13^

It is estimated that about 83% of the SA population use public healthcare facilities but only 30% of doctors are employed in the public health sector, resulting in an inequitable distribution of resources.^14^ The quadruple burden of disease (HIV-related disease and TB, interpersonal violence and trauma, maternal- and child-related health problems and non-communicable chronic diseases), poverty and unemployment and limited access to housing and sanitation for the poor are factors that further contribute to the challenges faced in PHC settings in the public sector in South Africa.^14^ Challenges faced in PHC settings have been associated with high levels of burnout, stress and anxiety.^5^ These challenges include high levels of uncertainty related to treating undifferentiated patients, no time for recovery from difficult clinical encounters, little support from management and working with disadvantaged or marginalised individuals.^10^

The strategy of improving healthcare delivery by the SA government is centred on the gradual introduction of a national health insurance (NHI) and re-engineering of the PHC.^15^ The aim of NHI is to offer universal, equitable and affordable healthcare coverage to all South Africans.^16^ Strengthening of the PHC service is a fundamental step in achieving NHI. Family physicians (FPs) play an integral role in improving PHC.^17^

Family physicians are expert generalists who have completed a full-time 4-year postgraduate training course in a Master’s of Family Medicine programme, which is a relatively new speciality in South Africa. Family physicians in the public sector work within the district health system (DHS) and are employed for part of a sub-district at a district hospital or community health centre (CHC), where they are the senior clinicians and play an important role in providing holistic comprehensive primary care with a broad set of skills and competencies in an increasingly resource-constrained environment in close partnership with a multidisciplinary team.^18^

The key roles of an FP within the SA context have been described as a care provider or clinician, consultant, capacity builder, supervisor, manager, community-orientated primary healthcare (COPC) champion and a leader of clinical governance.^18^ In practice, their roles include not only training undergraduate medical students, interns and registrars during the course of clinical service, but also capacity building of other health staff. Clinical governance in the form of audits, quality improvement cycles and regular morbidity and mortality meetings forms part of the day-to-day functioning of an FP in PHC in the DHS.

It is clear that the role of FPs in South Africa is multi-faceted and loaded. The expectations regarding clinical competence, mentoring, teaching, management, research and stewardship could potentially further contribute to burnout. As FPs balance these different roles and strive to meet the needs of the SA district healthcare system, they are particularly vulnerable to burnout.^19^

Despite the high prevalence of burnout in South Africa, and particularly in the Western Cape province, international studies have shown that some doctors and nurses who choose to remain in PHC survived, even thrived, despite stressful working conditions.^20,21,22,23,24^ The concept of thriving dates as far back as to the Greek philosopher Aristotle’s’ *eudaimonia*, which literally means ‘to have a good spirit’.^25^ Modern psychology has interpreted it to mean human flourishing.^23^ Why do some doctors manage to effectively cope with adversity and others not? Studies have shown that high levels of resilience among doctors are associated with decreased burnout, increased job satisfaction, a higher tolerance of both general and clinical uncertainty and a feeling of accomplishment.^6,22,24,26,27,28^

The definition of resilience varies in the literature.^20,26,29^ Resilience has broadly been defined as ‘a dynamic process encompassing positive adaptation within the context of significant adversity’^10^ or ‘personal qualities that enable one to thrive in the face of adversity’.^30^ Most researchers agree that resilience is situational, contextual and multidimensional rather than something that is fixed or static and can be learnt over time.^26,29,31,32^ Resilience has been proved to protect against burnout.^6,24,28,32^ A recent international review on resilience in PHC professionals found that ‘resilience in the professional is represented by continuing to perform well, adapting to changing circumstances and maintaining a sense of professional and personal fulfilment’.^26^

While systemic ‘top-down’ strategies at the policy level that aim to support improved working conditions for doctors and retain experienced doctors for prevention of burnout^6^ are needed, it is difficult to bring about this change which may take a long time. It is believed that amongst the pool of long-term employed FPs in PHC, there may be some who display certain characteristics or behaviour which promote resilience.^33^ By understanding what works for individual FPs through a bottom-up approach of personal positive reflection, rather than focusing on the deficits within the system,^33^ we hope to contribute to strengthening resilience. The retention of motivated, engaged and experienced FPs may be an important albeit one strategy in improving the quality of PHC. It could potentially contribute to bring about a change within the culture of medicine, and ultimately in the healthcare system.^6,24^

There is a paucity of local research exploring resilience and the factors that keep FPs working in a stressful environment such as the PHC settings. This research aims to explore the understanding and experience of FPs with regard to resilience. The objectives of this study include assessing the level of resilience in FPs working in PHC for more than 5 years, to explore their understanding of resilience, to identify attributes that contribute to personal resilience, understand the strategies used to manage stressors and to explore work satisfaction and their motivation for staying in PHC. The emerging information could assist efforts to ensure that PHC doctors are appropriately supported to deliver high-quality care.

## Aim

To explore resilience in FPs working in PHC in the Cape Metropole.

## Objectives

The objectives of this study were:

1. to assess the level of resilience in FPs working in PHC
2. to explore the perspectives and understanding of FPs regarding resilience.
3. to explore attributes that contribute to personal resilience and the coping strategies used to manage stressors
4. to explore the work satisfaction of and reasons for FPs to stay in PHC.

## Methods

### Study design

This phenomenological qualitative study was conducted using semi-structured interviews. A qualitative research design was chosen as this was deemed the best way to obtain an in-depth understanding of the experience of FPs working in PHC regarding resilience.

### Study setting

This study was conducted among FPs working in PHCs in the public sector of urban Cape Town metropole district of the Western Cape province in South Africa. The Cape metropole is one of six sub-districts of the city of Cape Town. The Western Cape was estimated to have a population of 5 755 607 in 2011 and approximately 83% of the population was dependent on public health services.^34^

### Selection of the study population

Non-probability purposive sampling with snowballing was used to select FPs. Family physicians who had a reputation as someone who seemed to cope with the challenges and display resilience in the workplace were contacted. These FPs could then nominate other FPs who would be possessing the same characteristics. Inclusion criteria were as follows: FPs who were permanently employed in the public PHC setting, namely, a CHC or a district hospital in the Cape Metropole, for more than 5 years and who may or may not work overtime.

The exclusion criteria were as follows: FPs who have been working in PHC for less than 5 years, those who are in the private sector or in secondary, regional or tertiary healthcare, FPs working outside of the Cape Town metropole area or FPs who are employed on a part time basis, for example, as locum tenens.

### Data collection

Potential participants were invited to participate in the study via email. The aim and objectives of the study were explained to them. They were requested to complete an informed consent form, a demographic questionnaire and a validated resilience scale which was then returned to the researcher via email.

The Wagnild and Young resilience scale (RS), a validated assessment tool, measures global resilience that comprises five characteristics: perseverance, equanimity, self-reliance, meaningfulness and existential aloneness.^31^ The RS has shown to have internal consistency and reliability (Cronbach’s alpha of 0.85–0.94). It contains 25 items, all of which carry a seven-point Likert scale. A higher score corresponds to a higher resilience. Scores greater than 145 indicate high resilience, scores of 125–145 indicate moderate levels of resilience and scores of 120 and below indicate low resilience. The resilience score was measured to have an objective measure of the participant’s perceived level of resilience.

Eligible participants were then invited to participate in a semi-structured in-depth face-to-face interview. Those who agreed to participate signed a second informed consent form. Each interview was conducted in private room agreed mutually, in English and lasted for about 20–55 min (average 32 min).

An interview guide was used and volunteered responses were further explored. The interviews were audio taped and field notes were made by the researcher. The interview guide was piloted to assess the validity. Participants were encouraged to reflect on the following definition: ‘Resilience is the ability to maintain personal and professional wellbeing in the face of on-going work stress and adversity’.^29^ Further probing questions were asked.

Because this was a qualitative study, the researcher planned to conduct about 8–12 interviews using snowball sampling. The interviews stopped when a point of data saturation was reached and no new themes emerged. A transcriber performed verbatim transcriptions of the audio recordings. Themes were inductively derived from the analysis.

### Data analysis

All transcripts were checked for anonymity and accuracy against the original audio recordings and sent back to the interviewees for member checking before further analysis of the data was conducted. Participants were given the opportunity to correct any possible misinterpretations to ensure validation of the accuracy of the transcribed interviews. All demographic questionnaires, audio recordings and transcriptions were stored on a password-protected computer.

Thirteen audio recordings, verbatim transcriptions, demographic questionnaires and the reflective field notes of the researcher were analysed using the framework method of qualitative analysis.^35^ The following steps were followed:

- Familiarisation of raw data: The researcher familiarised herself with the transcripts and noted key ideas. These and any issues relating to the quality of the data were discussed with the supervisor.
- Formation of a thematic index: To develop a thematic index, the researcher derived codes inductively from the transcripts, defined the codes and organised them into categories which were aligned with the objectives of the study.
- Indexing: The codes were then applied to all the transcripts using ATLAS.ti (version 1.6.0) software.
- Charting: Using ATLAS.ti software, all data related to a particular code or category were collated to create a chart or report.
- Interpretation: The charted data were analysed and interpreted to understand the nature and range of opinions and make associations or contradictions within the data. Quotations from the interviews were used to illustrate key points.

Interviews were continued and coded until thematic data saturation was reached.

### The role of the researcher

The researcher is a female family medicine registrar working in the Cape Metropole at the time of this study and is aware of the challenges encountered daily. Being an optimist by nature, with a conservative Christian worldview, the researcher felt fairly disheartened by the findings of a study that doctors in PHC were suffering from burnout and depression.^6^ The researcher questioned why some doctors managed to effectively cope and others not. This study was conducted with the aim that some insights could be gained from those FPs who continued working under stressful conditions.

The researcher was cognisant of the responsibility to accurately represent the views of the participants without being influenced by her own experience of PHC. Being aware that the participants would be sharing personal experiences, the researcher used attentive, courteous behaviour and a conversation style that helped to build a rapport during the interview. Constant critical self-reflection by the researcher was needed throughout the interview and analysis phase to control potential bias and predispositions.

### Ethical considerations

Ethics approval was obtained from Stellenbosch University Health Research Ethics Committee (Reference number: S16/07/138) and permission was obtained from the Metro District Health System (MDHS) of the Western Cape province to conduct this research study. Participation was voluntary, anonymous and by means of informed consent.

### Results

Thirty-three potential FPs were invited to participate via email. Ten FPs did not respond (30%), four declined participation (12%) and six did not meet the inclusion criteria (18%). The RS was completed by 15 FPs; however, two of those FPs did not meet the inclusion criteria.

Thirteen participants, six women and seven men, between the age of 38 and 57 years (average age: 46 years) fulfilled the inclusion criteria and participated in the study. Thirteen interviews were conducted between May and November 2017. The average duration of working in PHC was 14 years (range: 5–25 years). All participants were South African citizens who completed both their undergraduate and postgraduate medical training in South Africa. The majority of participants were married (76%) and had children (84%). The mean RS was moderate at 136 (range: 94–164).

Six major themes emerged from the interviews. The themes relate to key aspects of resilience which are further discussed.

### Sense of purpose

The majority of FPs were working in PHC even before family medicine was formally recognised as a speciality in South Africa. Typically, FPs started working in PHC early on in their career, or returned soon after completing their compulsory community service year. Family physicians stated that they were attracted to PHC during their undergraduate medical training and this interest was generally further piqued by role models or through the support of a mentor who encouraged them to pursue family medicine as a career:

‘There was a mentor who encouraged me to go and study family medicine. She was an amazing woman, that was when I worked in Limpopo. And then when I started studying family medicine, I met all these amazing teachers.’ (FP 6, Female, 48)

Most FPs are driven by a sense of calling towards their career and purpose in life which contributes to their ability to handle the stress associated with their profession. Providing quality healthcare to those who need it most gives them a sense of satisfaction and adds value to their lives. Family physicians feel that when they can make a meaningful contribution, they are needed. Others have a strong sense of duty to ‘give back’ to those communities who are most vulnerable and marginalised. The violent crime and social discord so prevalent on the Cape flats have a direct effect on the healthcare workers. Despite a serious threat to their personal safety and being victims of crime, certain FPs have remained working in the very community where these events occurred. Similarly, the industrial action strikes have not deterred certain FPs from continuing to serve these communities:

‘So for me it’s very important to have job satisfaction, and I think that also helps you to be resilient because you’re enjoying your job and you’re being – you’re getting rewarded, feeling like you’re adding something, you’re contributing, what you’re doing is worthwhile, then you’ll feel better about everything, no matter what the stresses, it’s worth it because you are making a difference.’ (FP 3, Female, 43)

‘… [*R*]ealising that the chaos in the [*Emergency Centre*] each weekend, and all the admissions and the amputations and the uncontrolled, this, that and the next thing. But, it is all sitting in PHC. And so you learn more and more that if you really want to make meaningful interventions, it is out there [*in PHC*] that we actually have to make a difference, or that we can make a meaningful difference, not just putting out the fires, but really getting in there. So, for me, PHC is where the need is, and where we can make a significant change to the health care.’ (FM 10)

Those who entered the work space with a clear vision of what they wanted to accomplish as an FP, or in their personal lives, considered this a contributing factor to their resilience, as this concept kept them focused on their goal regardless of the many demands placed on them. In addition, the feeling that their ideas and contributions are valued, either by colleagues or the patients they treat, aids in their desire to stay in their profession:

‘When we voted in 1994, we voted for a better life for all. And all South Africans, all human beings deserve good medical care when they need it. So the majority of South Africans can’t afford private care, so I’ve always wanted to work where I’m needed, and I’ve always felt a lot more [*is*] needed in the public sector.’ (FP 6, Female, 48)

### Silver lining thinking

All participants felt that their perception of and attitude towards their work had a significant impact on their ability to handle the daily challenges. Family physicians viewed their work as being of importance beyond themselves. Their attitudes towards serving the community, making a difference or even seeing their work through the lens of their particular worldview helped them to persevere despite adverse conditions.

It was clear that through self-awareness and reflection, FPs recognised and accepted their own strengths and limitations. They had realistic expectations of what they could and could not change in their environment. As such, they tend to have an optimistic philosophy of life and preferably spend their energy on what can be influenced:

‘… I will try to put things into perspective and just see what it is that can be done, and then often just try to do with the best you can, because often you can’t solve the problem, but instead you can do the best that you can, then the best thing can be satisfying. People are maybe unhappy – that they can’t solve the problems but sometimes the best is not to solve it but just to do the best you can.’ (FP 2, Male, 48)

While often being surrounded by extremely negative conditions as a result of poor socio-economic conditions in South Africa, these resilient FPs rather focus on the positive stories by celebrating the patients and programmes that do well as opposed to constantly fixating on all the discouraging situations they encounter daily. This is not to say that they are naïve and ignore the negatives, these are dealt with if and when the opportunity is right:

‘And we need to start moving towards more of a solution-focussed, interactive approach where we start you know focusing on solutions and focusing on where we can actually help each other. Ja, you know so you, you may be faced with a problem in the community of TIK (crystal methamphetamine) and psychosis or gangs you know, gang violence but the question is you know how are we really engaging the communities, are we drawing on the resilience of the communities and the individuals around you that can actually help your resilience.’ (FP 4, Male, 53)

Family physicians recognise the need for self-awareness and reflexivity, as they are encouraged to take the time to reflect on their personal and professional experiences. This is an aspect of their speciality which some FPs particularly value as it is not overtly encouraged in other specialities:

‘Self-reflection in family medicine is a big part of family medicine, compared to other specialities where they don’t encourage reflection on why did you react like that at that situation.’ (FP 9, Female, 38)

‘I know others may say we’re too fluffy, but it does make a big difference. It makes a difference to resilience and how you’re coping, to know why you chose, whether it’s clinical or whether it’s management situations, why did you choose that path and not another path? And you’re going to explain yourself and answer to yourself, and it’s resilience at the end of the day, to say I chose that route and I stand behind it, because I thought it through, and on reflection I realised that that was the best option.’ (FP 9, Female, 38)

Engaging in the practice of mindfulness appears to be a successful way in which these FPs are able to cope with the everyday stressors. Being present in every moment, whether in professional or personal capacity, assists the FP to cope:

‘And you need to be in every single moment … one of the nicest things that the mindfulness experience has taught me is that if you manage to be happy in the moment, now, so there where you are sitting now, if you are happy now, you will always be happy. Okay, so if you ensure that every moment, whether you’re at work or at home or giving a seminar, whatever, if you manage to make sure that in that moment you’re happy…’ (FP 11, Male, 48)

### Several roles with autonomy

It was apparent that most FPs have remained in PHC because they feel most comfortable and enjoy the facets unique to PHC. Family physicians welcome the mental stimulation derived from attending complex undifferentiated complaints and tend to tolerate diagnostic uncertainty. The opportunity for community involvement and the capacity to make a positive impact on disease outcome through health promotion and disease prevention strategies are other facets which FPs embrace:

‘[*FPs*] tolerate uncertainty and you know, almost relish uncertainty because uncertainty is what we’re good at. If you bring specialists into a low certainty environment, they would just freak out. They wouldn’t last a day. Because what they need in order to reduce their stress levels is lots of diagnostic tools which we don’t have. We have our clinical acumen and our feel and our ability to be empathic and to engage with the whole patient, and all of that. We have that. Those are the tools that we … and we’ve got to enjoy that.’ (FP 8, Male, 57)

‘I have worked with a lot of [*physicians and surgeons*] and you realise that by the time the patients reach the hospital level, you have usually missed the boat, you know as soon as they come to [*hospital*] and you have got the amputations, they have actually missed all the opportunities that you have had in primary health care … so I am passionate about you know health promotion, and prevention and trying to keep people well you know rather than doing like high tech stuff at a hospital level.’ (FP 4, Male, 53)

All FPs interviewed concurred that their job inherently entails a variety of duties. Interestingly, FPs seem to thrive in the diversity and reflected that their various responsibilities and different roles bring a sense of relief and tend to make the volume of work more manageable. Those FPs who felt that they had more autonomy and control of their workload felt more resilience and were objectively more resilient:

‘I am staying here because I do get some time off that I can take to teach and get students and do the online teaching and do the exams … the fact that my work has got lot of aspects; there’s admin, there’s management, there’s seeing patients, there’s teaching, there’s research and exams and so there is lots of aspects to the job. Which makes it nice and that’s one of the reasons why I am staying, if I just had to see patients every day, forty patients every day I wouldn’t have made it you know. I would have left long ago so that’s one of the reasons why I am staying.’ (FP 7, Female, 45)

‘I am a generalist and I actually enjoy doing a little bit of everything you know, I enjoy being able to go to theatre and then you know to be going to the EC and counselling someone that’s taken an overdose and then to run off to a management meeting and then on another day to go to another clinic.’ (FP 4, Male, 53)

Although PHC offers a diversity of ‘cradle to grave medicine’ over time, some FPs have found a niche for themselves within the broader scope of PHC. Many of these FPs find that their individual passions pull them towards certain elements and duties within the variety:

‘So I think that also helps. You know where you are able to match your, your personal passion with, what you really, you know really interested in, where you can focus some time. Dr X for example, loves obstetrics, he loves PHC teaching, you know so he is been more and more involved with that. And he also has a flare for management.’ (FP 4, Male, 53)

In contrast, others admit that having competing demands can be overwhelming at times and difficult to juggle. Family physicians expressed that they were likely to be more dissatisfied when they felt out of control or were not able to meet the various demands placed on them by different authorities. In addition, tension exists between certain roles, especially as a leader of clinical governance versus being a competent clinician. Governance functions aimed at quality improvement conflicted with the need to meet the clinical demands of a high patient load. Some participants felt that the FPs ‘should jump in and help’, while others felt that by doing so constantly the systemic problems or ‘system stuff’ are neglected or even perpetuated. At the same time FPs recognise the need to be flexible and adapt to the needs of their facility:

‘[*I*]f you just roll up your sleeves and diving in there and helping out on the floor because it’s busy today, you’re not really going to be doing your job. And in the end you’re not really contributing to, you know? But we all feel really guilty about pulling ourselves out of it. And then even when you do pull yourself out of it and continue to do what you’re supposed to do, it’s just too much.’ (FP 5, Female, 50)

Medical officers are expected to see approximately 40 patients per day, straining the time spent with each patient. Family physicians in contrast feel less pressure ‘to push numbers’ and as such they are able to offer more comprehensive and holistic care to patients. As such, while most FPs were considered to be resilient, they expressed concern for junior doctors:

‘So I can be resilient but I, you know I worry a lot about our junior doctors and their conditions, and it’s not always in my control to improve it, but I would want to improve it. Because I think you can only be so resilient but I think the working conditions have to be sustainable.’ (FP 12, Male, 45)

### Skilful leadership

The majority of FPs shared that effective leadership was an important contributor to resilience. Family physicians feel that a leader should work as part of the team, be willing to lead and follow where appropriate:

‘You need to build a good team, you need to be a good team leader, a good team player to identify areas that people are passionate in and genuinely interested in.’ (FP 4, Male, 53)

All FPs were of the opinion that fostering communications skills and good relationships amongst all levels of staff were important to accomplish work successfully. As leaders, FPs need to be intentional about creating a pleasant working environment where discussion or project participation allows for the development of these relationships:

‘I think a lot more effort needs to be put into allowing staff to kind of engage and talk to each other, as opposed to just come to work, you see your patients, you go home.’ (FP 5, Female, 50)

The FPs agree that in order to be an effective leader and meet the demands and workload, strategies such as delegating tasks to the team, prioritising and setting personal and professional boundaries are essential. These strategies were seen as a way of cultivating personal resilience. It was also felt that one needed managers and colleagues who recognised and respected those boundaries:

‘I think for me, you know in primary care, you know having a multifaceted role actually helps; it helps in resilience because it gives you a bigger picture and you get involved in different parts of the team. You are actually acting as a conductor in an orchestra. And you know part of your, your roles is actually you are not actually sitting in the front line doing all the work. You are identifying people that can actually help you.’ (FP 4, Male, 53)

Resilience is seen as a characteristic of those who have successfully implemented changes contributing to a more efficient system, or those who have made a positive impact on their work environment. Some FPs stressed the importance of becoming familiar with the demands of their working environment, and the difficulties experienced by team members before instituting any changes. Valuing inputs from the entire team, and respecting the specific culture of that institution ought to be understood before changes are implemented. Flexibility in the approach of FPs to their responsibilities is an essential skill necessary for resilience:

‘You can come here with the fanciest of ideas and models and that, unless you speak to people, to make them understand why we’re moving in a certain direction, and find out why there are challenges. And it took us years. I mean a lot of the stuff that we’ve got going now, I mean for example the TB, a lot of things that we tried to do over the years, failed dismally, and it failed because we – we came in thinking that we’re just going to change things, and we learnt the hard way. And with time you sort of build those relationships, and it’s all about those relationships.’ (FP 1, Male, 46)

‘So that has helped, just having a flexibility in terms of what is needed at the time. For me one of my key things that I see myself being responsible for is making sure that the staff are happy.’ (FP 3, Female, 43)

### Support networks

Family physicians felt that cultivating relationships with one’s spouse, family or close friends outside of the work environment was a vital contributing factor to well-being. They not only offer support and understanding but also provide valuable perspectives. Most FPs recommended that work should not interfere with family time and that existing bonds should be protected. Most FPs try to separate work life from home life by assigning a fixed time for the latter; however, some admitted that because of the immense workload, work encroaches on home life at times:

‘And often I go home and talk things through generally with my husband, but sometimes with my parents, and then come back to work with a new idea of how to approach a problem, and it’s having talked it through with other people.’ (FP 9, Female, 38)

Support from colleagues was seen as essential to enable open communication, a good working relationship and team work. Participants expressed that support from not only colleagues at the facility but also those at the referral centre and the community was important:

‘I think one of the most important things in the work place is to maintain a good, good working relationship with your team and with your colleagues … we make a point regularly of meeting every day and meeting for tea, meeting for lunch, and also collegial support is actually very important.’ (FP 4, Male, 53)

There should be a network of support from other FPs to exchange honest views and experiences and share information, knowledge and skills. This contact is fostered by the Family Physicians Forum, which is attended by all FPs in the Metro District Health Services (MDHS):

‘… [*T*]here are senior FP colleagues that I have who I can just talk to and say this is what’s bothering me, I’m thinking of doing this, what do you think, and they can assist me with that, so it’s just the personal support that I think helps me.’ (FP 12, Male, 45)

Family physicians tend to feel more supported by their managers when their role is clearly defined and expectations are openly communicated. In addition, situations when FPs are given the time to attend to the competing demands or given more time to spend on certain projects also contribute to resilience. On the other hand, FPs perceived that most managers do not always know the role or function of the FP. This could at times result in FPs being expected to perform duties which are not their responsibility or part of their job description:

‘[*My manager*] has created the right environment when it comes to expectations where I work and my job description, and we’re fairly on the same page, most of the time, so it helps that there’s not that expectation gap. Which there sometimes is, but good communication helps that, but I think I’m supported in what I’m trying to do, and I’m getting the time to do those kinds of things.’ (FP 12, Male, 45)

Professionally, FPs recommend having an older or more experienced colleague whose opinion one values and one who offers advice. Mentors offer wisdom and vast experience in not only clinical aspects of work but also relational aspects:

‘… [*Mentor*] has been a wise person, some years back I really struggled to figure out, how do we work with the nurses, and his advice to me at one of the conferences, we had this conversation, he said get everyone to focus on a common goal. The goal is patient care. You know, it’s not us against them, the doctors against the nurses. It’s how do we work together, towards a common goal. And over the years that’s what I’ve been trying to do with working in teams. But it’s not about the personalities and the egos and what you did wrong, it’s about, what is the best that we can do for the patient.’ (FP 6, Female, 48)

In addition, the value of coaching was emphasised. Most FPs had benefited from individual or group coaching sessions either provided by the Department of Health or by the Stellenbosch University’s Department of Family Medicine and Primary Health Care:

‘… [*T*]here is great value in, in enlisting the help of a professional coach. With the help of Stellenbosch University we had a professional coach. We had individual coaching sessions. We had group coaching sessions. That was also helpful. I think personally for me, you know especially in primary care when you are multitasking and the work environment is sometimes chaotic, you know I have realised the importance of actually getting organised and being disciplined around planning.’ (FP 4, Male, 53)

### Self-care

Family physicians recognised the importance of practices such as getting adequate nutrition, sleep, regular breaks and leisure-time activities aimed at ‘honouring self’ in order to provide optimal patient care:

‘Because one needs to also value yourself enough to care for yourself, in order that you’re able to care for the patients. And that means having lunch, getting enough sleep, exercise, which for me is walking the dogs, but it’s to have a healthy lifestyle as a self-care thing, because one needs to have that balance that you can’t just be a slave to the system. You’re going to burnout. So you need to look after yourself so that you can keep functioning in the system.’ (FP 6, Female, 48)

Interest outside of medicine was found to be a source of pleasure. Leisure-time activities include sports, music, art and literature. One FP learned a new skill in order to address the recognised burnout as it allowed a change in mental focus:

‘There was a time 2 years ago when things were difficult, and I realised I need to do something to look after myself; I started music lessons. So I played the Alto Recorder. So on Tuesdays I leave work at 16:30 so that I can be at my music lesson at 17:00. And that once again stimulates my brain in a different way.’ (FP 6, Female, 48)

Most FPs expressed that their faith or belief system gave their personal and professional life more meaning. This sense of purpose contributes to their resilience:

‘I have strong faith in God and I believe there is a bigger meaning to life, that is quite a significant factor in my life, and I believe it plays a role.’ (FP 10, Female, 38)

‘So I often speak to my patients about religion you know, although they’re Christian or from another denomination, to influence them in a way that despite having differences from a religious point of view, but just hold on to – holding on to what is important within that religion, and you know it stems, it all comes down to the faith, and the belief and all that.’ (FP 1, Male, 46)

A few of the FPs interviewed shared that they were currently or had previously been on medication for mental health issues related to burnout. Most FPs admitted to either coming close to burnout or experiencing burnout early on in their careers. But through the support of family, colleagues or professional, they persevered. Seeking professional help when needed was emphasised. Family physicians either sought private psychological or psychiatric help at their own cost or they contacted the Independent Counselling and Advisory Services (ICAS), an organisation which provides a confidential 24-h professional counselling service at no cost to employees of the Western Cape Department of Health:

‘So [*my supervisor*] recognised as well that I was battling, and I recognised it but there was some personal things happening at the same time, that was just culminating. And I took myself off to a psychologist. And [*I*] was with her every week for a year, and I think that being able to say I need help now is something that is important.’ (FP 9, Female, 38)

Contrasting views on work–life balance were expressed. While some intentionally strived to separate the two, some were of the opinion that this concept is a myth, and the attempt to find an ever elusive work–life balance further contributes to the sense of burnout. Instead, one ought to be mindful in every moment, seeking to fully embrace all experiences:

‘So this thing about life and work balance, it’s an illusion. There’s only life. And you have to balance your life. So this idea that it’s separate things that must be separated is part of the problem, because how do you divide the time? You can’t. And how do you decide which gets priority and so uh-uh, just kill that thought. There’s only life. And you need to be in every single moment …one of the nicest things that the mindfulness experience has taught is that is if you manage to be happy in the moment, now, so there where you are sitting now, if you are happy now, you will always be happy.’ (FP 11, Male, 48)

## Discussion

This study identified six key aspects of resilience in FPs working in PHC: having a sense of purpose, ‘silver lining’ thinking, having several roles with autonomy, skilful leadership, having a support network and valuing self-care.

Our study supports the well-established concept that the features that promote resilience are multi-factorial.^21,26,28^ We found that being resilient does not only depend on individual attributes, but also on the environmental aspects and, most importantly, on the relationships one has both at work and at home. This echoes the findings of Jensen and colleagues, who identified four main aspects: (1) attitudes and perspectives, (2) balance and prioritisation, (3) practice management style and (4) supportive relations.^28^ Likewise, a German study, conducted by Zwack et al. found that (1) job-related gratifications; (2) practices, such as leisure-time activities, self-demarcation, limitation of working hours and continuous professional development and (3) attitudes, such as acceptance of professional and personal boundaries, a focus on positive aspects of work and personal reflexivity all fostered resilience.^21^

The four participants who had a high RS score had a positive attitude towards their work and found meaning in their work. Family physicians had an overall feeling that they were ‘making a difference’ and reported job satisfaction despite the overwhelming workload. Similarly, in a systematic review of 20 studies on resilience in PHC professionals, Robertson and colleagues found that resilience enables the professional to manage workload demand with the assistance of external supports both within and beyond work. They further highlighted the importance of personal meaning or a sense of purpose, although it is not clear whether this drives resilience, or arises as a consequence of resilient behaviour.^26^ Our findings also reflect Warner’s principles to building resilience, which include the need to connect to the meaning in one’s life, self-awareness, maintaining perspective and dealing with negative thoughts and feelings, developing a realistically optimistic outlook, being open-minded and flexible and reaching out to others for support and help.^36^

Those FPs who believed they had more autonomy and control over their workload and projects felt more resilience and were objectively more resilient. This is in agreement with a study conducted by Keeton and colleagues who found that having some control over schedule and hours worked was the most important predictor of low burnout and work–life balance.^37^ Similarly, having autonomy, which involves self-regulation, accountability, clinical decision-making and advocacy, predicted lower levels of global burnout in nearly 900 physicians in Israel.^38^ Also, a study of 8050 physicians demonstrated that professional autonomy was more important than income in determining a physician’s career satisfaction.^39^

A systematic review and meta-analysis on studies for interventions to prevent and reduce burnout found that overall burnout was decreased by 10%. Both individual-focused and structural or organisational strategies could result in clinically meaningful reductions in burnout among doctors.^40^

Regardless of the available resources, it seems that resilience is enhanced by relationship building, by humane interaction and respect for each other. Having a support network and fostering good relationships appear to be the most important characteristics of a resilient doctor. Family physicians in the Cape Metropole echo the importance of teamwork and encourage more shared power so that people may feel included and have some input in decision-making; these are in agreement with the current organisational values.^41^ Gilson and colleagues found that stable governance structures and sufficient resources are not enough to ensure resilience, but effective leadership that promotes mindful staff engagement and social networks and relationships are needed to ensure resilience.^19^ This appears to be similar in our resource-poor public healthcare setting.

Although most of the participants were perceived to be resilient by their peers (through snowball sampling) and objectively had moderate levels of resilience, many of the FPs however did not consider themselves to be resilient. Most FPs admitted to have experienced or come close to burnout at some point in their careers. These findings correspond with those of Polachek et al.^42^ who found that a difference exists between how physicians perceive resilience in themselves and what they observe in their colleagues. Specifically, FPs may hold unrealistic and unachievable expectations for their own resilience.^42^ These unrealistic expectations are perpetuated within the professional culture of medicine which praises perfection, and unduly judges those who display vulnerability.^43^ This serves to further highlight the need for cultivating resilience among FPs.

Cultivating resilience can be done using individual and relational coping strategies. Family physicians are encouraged to practice self- reflection and mindfulness, gain leadership skills and develop a sense of purpose and meaning. Building good relationships, as well as having a mentor and coaching are also encouraged.

## Limitations

Some FPs may have under-reported their stress because of the desire to be perceived as highly capable and in control. The RS measure used is based on personal characteristics and does not assess social and workplace challenges, which can be an important part of professional resilience. Our study does not distinguish between the inherent and acquired factors of resilience or the relative importance of the six resilience themes.

## Recommendations

To cultivate resilience in FPs, a combination of individual and organisational strategies is recommended. Family physicians are encouraged to practise self-reflection and mindfulness, develop a sense of purpose and foster meaningful relationships. Resilience can further be strengthened by creating an environment within the workplace where relationships can be cultivated, leadership skills can be developed and professional autonomy would be respected.

## Conclusion

The aspects that contribute to resilience in FPs are multi-faceted. It entails having a sense of purpose, ‘silver lining’ thinking, having several roles with autonomy, skilful leadership, having a support network and valuing self-care. Our exploration of resilience in FPs in the Cape Metropole corroborates the findings of previous studies. To ensure physician wellness and improved patient outcomes, we recommend that individual and organisational strategies should be implemented in the absence of long-term policy changes.
